# Experimental study of the freeze thaw characteristics of expansive soil slope models with different initial moisture contents

**DOI:** 10.1038/s41598-021-02662-9

**Published:** 2021-11-30

**Authors:** Zhongnian Yang, Jianhang Lv, Wei Shi, Chao Jia, Chu Wang, Yong Hong, Xianzhang Ling

**Affiliations:** 1 Qingdao University of Technology, Qingdao, 266033 Shandong China; 2 Shandong University, Qingdao, 266237 Shandong China; 3 Pennsylvania State University, State College, PA 16802 USA; 4 Harbin Institute of Technology, Harbin, 150001 Heilongjiang China

**Keywords:** Natural hazards, Civil engineering

## Abstract

This paper presents an experimental investigation on the effect of freeze–thaw cycling on expansive soil slopes with different initial moisture contents. Clay soil from Weifang, China, was remolded and selected to build the expansive soil slope for the indoor slope model tests. A total of five freeze–thaw cycles were applied to the three expansive soil slopes with different moisture contents ranging from 20 to 40%. Variations of the crack developments, displacements, soil pressures and moisture contents of the expansive soil slope with different initial moisture contents during the freeze–thaw cycling were reported and discussed. The results indicate that higher moisture contents can slow the development of cracks and that the soil pressure increases with decreasing temperature. The soil pressure of slope decreases after freeze–thaw cycle, and the change amplitude of soil pressure after freeze–thaw is proportional to water content. The slopes with a moisture content of 20% and 30% shrinks during freezing and expands during thawing, which was named *ES-FSTE* Model, while the slope with a 40% moisture content shows the opposite behavior. During freeze–thaw cycles, moisture migrates to slope surface. As initial moisture contents increase, the soil heat transfer rate and bearing capacity decreases after five freeze–thaw cycling.

## Introduction

Over recent decades, extensive attention has been given to freeze–thaw cycling in expansive soil engineering applications, including construction of highway and railway^1,2^, control and protection of slope^3,4^, and treatment of engineering foundation^5,6^. Freeze–thaw occurs due to the phase change of moisture in the soil subject to external temperature variations. This process deforms the structure of the soil, and alters the mechanical properties of soil, which has induced a variety of disasters in engineering applications^7–10^.

Expansive soil are recognized by expansion, collapse, multicracks and overconsolidation; accordingly, numerous engineering accidents have been caused by the instability of expansive soil slopes, leading to severe consequences^11^. The factors involved in slope stability during freeze–thaw cycling in frozen soil areas are complicate^12^. The characteristics of slope stability changes under different freeze–thaw conditions need to be studied. Yang et al.^13^ found that there is downward creep tendency of expansive soil slope with large slope ratio during freeze–thaw cycle, which increases the risk of landslide. Kong et al.^14^ studied the expansive mudslide in the expansive soil area of northeastern China and found that seasonal moisture changes and freeze–thaw cycles were the main causes of the landslide. For the stability of unsaturated expansive soil slope, some scholars have also tried to propose calculation methods. Qi and Vanapalli^15^ presents a calculation method for slope stability of expanded soils that may be suitable for meltthawing infiltration, but this method only considers the effect of a single thaw on the stability of the slope and does not account for the continued destruction of the slope by the course of multiple freeze–thaw cycles. Some calculation models for stability analysis of expansive soil slope considering rainfall and seepage are put forward^16,17^, but they are also not applicable to the calculation of slope stability of expansive soil under freeze–thaw cycle conditions. Tests are required to provide effective basis for long-term stability of expansive soil slope. Several studies have proposed methods to control the stability of expansive soil slope, including crack filling^18^, soil bag reinforcement^19^, artificial drip irrigation^20^, etc. However, the effectiveness of the above treatment methods for freeze–thaw expansive soil slope is unknown.

During the freeze–thaw cycle, the physical and mechanical properties of fine-grained soils such as expansive soils change dramatically. Freezing–thawing cycles change the dynamic properties of soils. Cui et al.^21^ finds the maximum dynamic stress a silty clay increases after freezing and thawing. Lu et al.^22^ investigates cracking behaviors in clayey soils under cyclic freezing–thawing and found crack pattern evolves from an irregularly rectilinear pattern towards a polygonal or quasi-hexagonal one. Yang et al*.*^23^ studied the dynamic shear modulus and damping ratio of frozen-thawed expansive soils and found that freeze–thaw cycling causes additional shear deformation of expansive soils, thereby increasing the dynamic shear modulus of the soils. At the same time, with the development of freeze–thaw cycle, phase change of ice-water will cause large displacement of soil. Dash^24^ believed that the nature of frost-heaving is that the pore pressure generated by ice crystallization is greater than the upper soil pressure, and as temperature decreases, the pore pressure gradually increases, the effective stress between soil particles gradually decreases to 0, and the formation of ice lenses leads to frost-heaving. At present, research on frozen soil melting and settlement is mainly based on the statistical relationship between frozen dry density and moisture content to obtain the melting and settlement coefficients of different soil^25^, and some scholars have gradually established a three-dimensional theoretical model of large deformation of melting settlement of frozen soil based on the consolidation principle^26–28^. However, frost heaving characteristics of expansive soils are different from that of common fine-grained soils. Luo et al.^29^ proved that in addition to the volume change caused by water ice phase change, there is also an increase in the volume of expansive soil particles caused by the increase of water content in the unfrozen area.

In actual frozen soil engineering, which is affected by construction, climate and other external factors, the soil moisture content is the most common variable. Luo et al.^29^ found that the freezing point of expansive soil will increase with the increase of initial moisture content. Lu et al.^30^ measured the mechanical degradation of three expansive soils with different initial moisture content during freeze–thaw cycle. It was found that the freeze–thaw damage of expansive soil was more serious under high moisture content. At the same time, it was found that the expansion and contraction trend of expansive soil under high moisture content was opposite to that of expansive soil with low moisture content. There is obvious moisture migration during freeze–thaw cycles^31^. The moisture content of the upper soil will increase to some extent after a freeze–thaw cycle. This is mainly due to moisture migration to the frozen front during the freezing process, which tends to make the moisture distribution in the frozen soil uneven. Therefore, the influence of the initial soil moisture content cannot be ignored^32^. Research on the stability of slope models under freeze–thaw cycling requires high-performance test equipment, and there are few studies on the stability of expansive soil slopes considering different moisture contents. Therefore, an experimental investigation on the expansive soil slope stability under different moisture contents would represent an important advance in the frozen soil engineering field. Experimental data obtained from such tests are often valuable for validations of analytical and numerical models and predications of in situ design. Moreover, the failure mode revealed in the laboratory experiment can be also very helpful to direct engineering design in the real world.

This paper takes the initial moisture content and freeze–thaw cycles of expansive soil slopes as variables during testing. The stability and internal characteristic changes of the expansive soil slope under freeze–thaw action are recorded by temperature, humidity, displacement and soil pressure sensors installed inside the slope. The mechanical characteristics of the slope after freeze–thaw cycling are observed by a loading test to reveal the internal variation in the expansive soil slope during freeze–thaw cycling and the bearing capacity after freeze–thaw cycling. The bearing capacity characteristics provide a damage model and design basis for preventing and mitigating expansive soil slope disasters in actual seasonal frozen soil areas.

## Experimental methodology

### Slope model preparation

*MeicuTown* clay expansive soil from Weifang, China, was remolded and selected for the slope models in this study. The basic physical properties of the selected expansive soil are shown in Table 1. The moisture content and maximum dry density of the soil samples were tested in accordance with the ASTM D698^33^.

**Table 1:** Mechanical properties of the selected expansive soil.

Natural moisture content (%)	Maximum dry density γ_dmax_ (g/cm^3^)	Optimal moisture content ω_opt_ (%)	Free swell ratio, *FSR*
9.14	1.53	20.17	1.51

**Figure 1:**
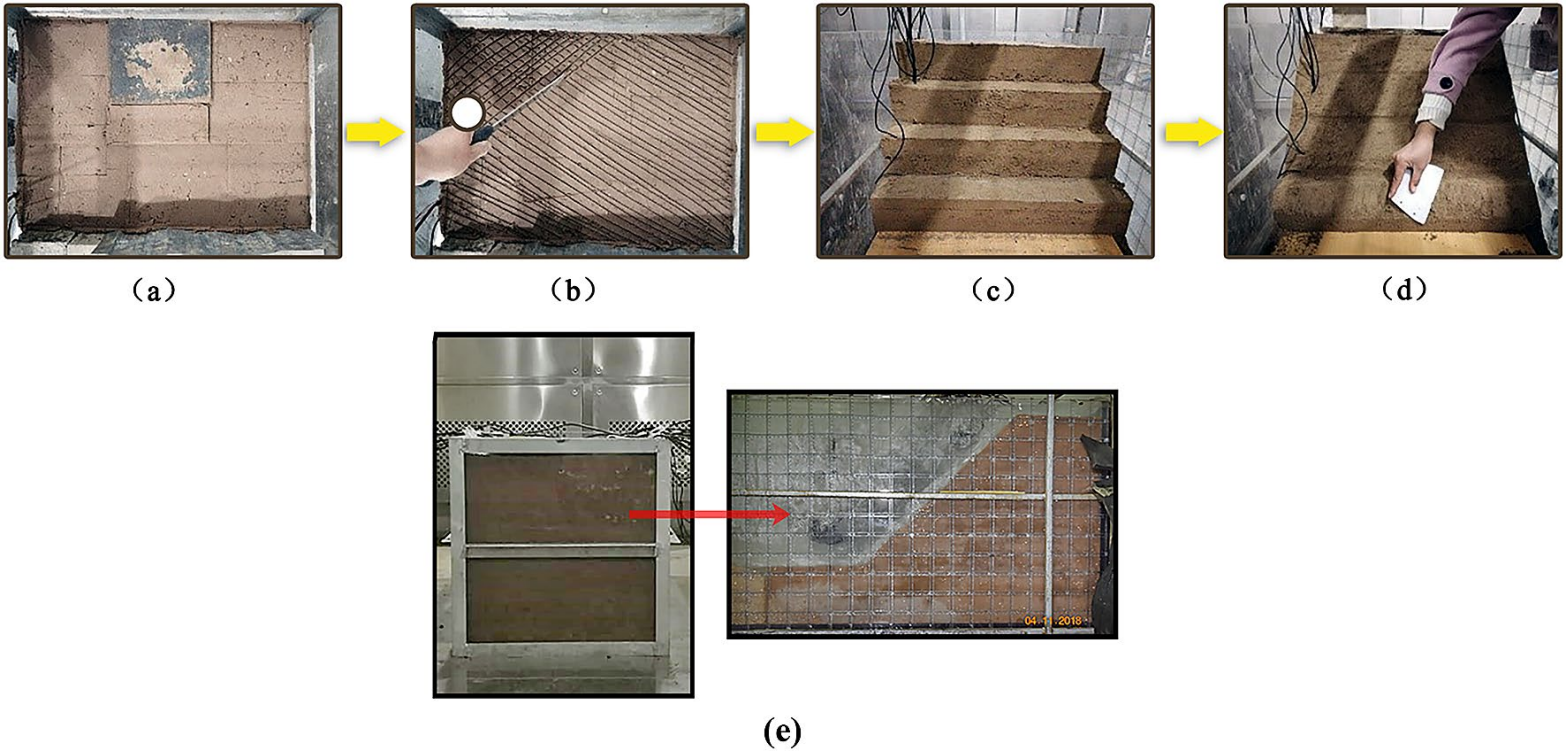
Slope preparation process ((**a**) layered compaction; (**b**) surface scraping; (**c**) dismantling support; (**d**) slope cutting; (**e**) finished preparation).

The expansive soil had a free swell ratio of 1.51 and was classified as moderate expansive soil respect to the classification criteria proposed by Prakash and Sridharan^34^. The expansive soil slope models had designated moisture contents of 20%, 30% and 40%, which are denoted $S_{20}$, $S_{30}$ and $S_{40}$, respectively. The expansive soils with different moisture contents were compacted in layers to form a slope model with a compaction degree of 79%. The model preparation process was divided into four stages, as shown in Fig. 1.

**Figure 2:**
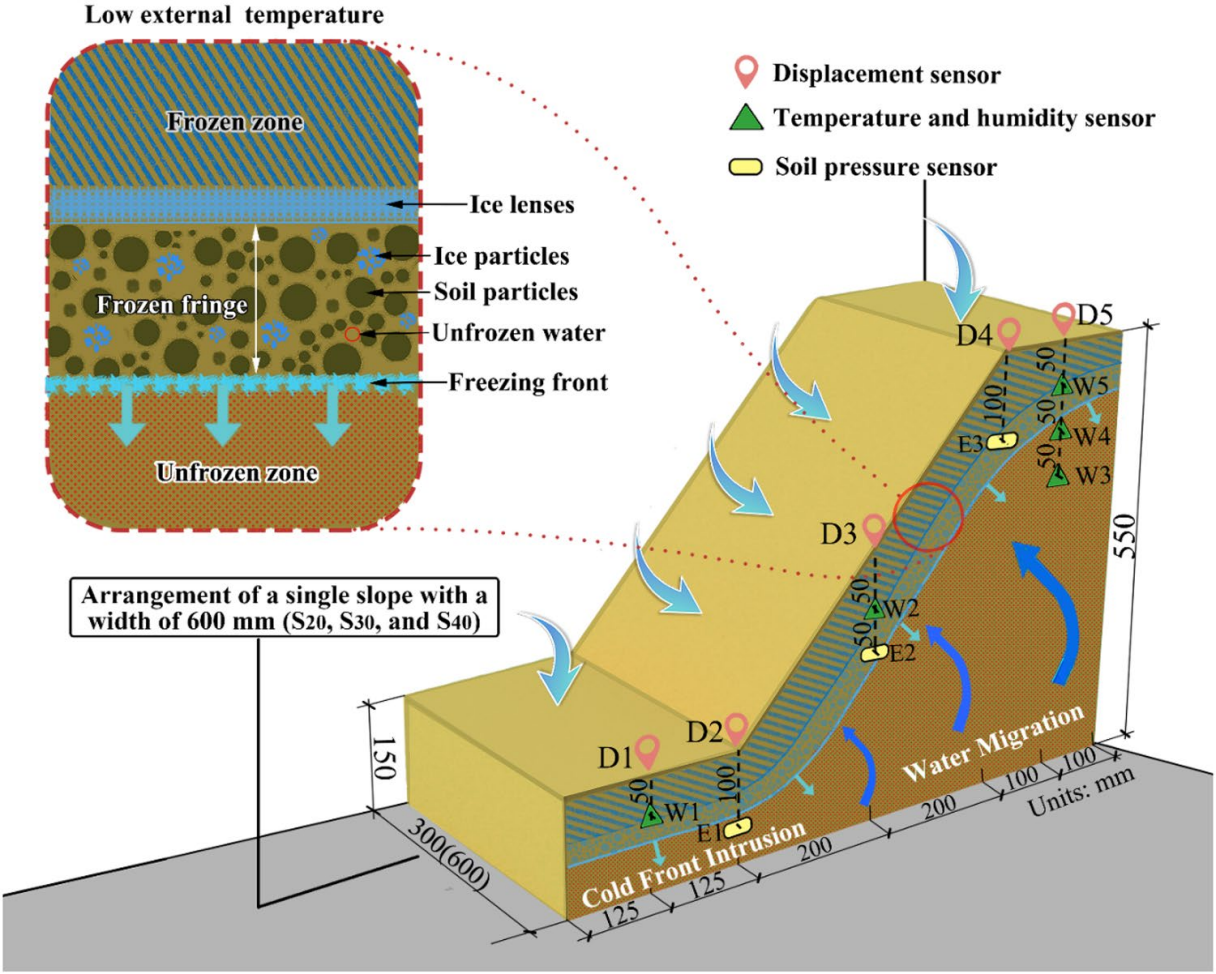
Slope freezing model and freeze–thaw monitoring system.

A large area of expanded land was found in the Yanji railway construction in Northeast China. In the excavated cut slopes, the expanded soil slopes received severe seasonal freeze–thaw effects. In this trial, referring to the cut slopes excavated at a local site, the design model had a similar ratio of 1:20. The dimensions of the slope models were 85 cm (length) × 60 cm (width) × 55 cm (height), the length of the top platform was 20 cm, and the slope ratio was 1:1 (Fig. 2). The three different expansive soil slope models were tested side by side. After the preparation of the slope models, the surface of each slope was covered with a multilayer film to ensure that the slope did not lose moisture without being influenced by heat transfer.

### Sensor layout

In each test, the changes in the displacement, soil pressure, temperature, and moisture content of the slope models were recorded during freeze–thaw cycling. The monitoring system used in this study is shown in Fig. 2. The displacement was monitored by an HG-C1100 (160 mm range and 70 µm accuracy) micro laser displacement sensor and LT-150-R displacement sensors, denoted as ***D1–D5***, which were arranged on the slope surface from the bottom to the top of the slope. The soil pressure was measured by DMTY resistance strain earth pressure boxes (0.05–10 MPa range), denoted as ***E1–E3***, which had a depth of 10 cm and were arranged along the slope from the bottom to the top, as shown in Fig. 2. The temperature and moisture content were measured with one integrated sensor (HM-S485), for which the measuring range of moisture content was 0–100% and the measuring range of temperature was − 40 to 80 °C. Moisture sensors can accurately measure the water content of unfrozen water in soil during freeze–thaw cycles, in which ice is considered solid rather than moisture in the soil. Five sensor groups were installed: ***W1***, ***W2*** and ***W5*** were arranged along the slope from the bottom to the top, whereas ***W3***, ***W4*** and ***W5*** were arranged along the depth of the top of the slope. During the test, the temperature, humidity, displacement and soil pressure of the slope bottom and top in the slope model are monitored along the vertical depth, so the sensor arrangement in the slope is shown in Fig. 2. During the preparation of the slope model, all sensors are arranged and manually compacted to ensure close contact with the surrounding soil. All signal transmission cables are assembled laterally and connected to the data analysis device along the horizontal direction without affecting the slope as much as possible. All sensors in the test operate within the set temperature range to ensure measurement accuracy.

### Experimental design

A custom-built multifunctional environment box was used to conduct the freeze–thaw cycling tests on the expansive soil slope models. For this test equipment, the temperature range was − 30 to 80 °C, the accuracy was ± 0.1 °C, the heating rate was 3 °C/min, and the cooling rate was 1 °C/min. Expansive soil is very sensitive to the freezing temperature. Hence, the effect of different freezing temperatures on the properties of expansive soil was investigated. The physical properties of expansive soil are most affected when the freezing temperature is sequentially set to − 10 °C and – 20 °C^35^. Therefore, − 10 °C and − 20 °C were selected as the freezing temperatures in this study. A constant temperature field could boost the slope to reach the designated temperature. After the slope freezes, the constant-temperature melting stage starts. Only when the absolute value of melting temperature is higher than the absolute value of freezing temperature can the soil reach a stable state rapidly^36^. Therefore, a melting temperature of 30 °C was selected. In all tests, the slope model was laterally cotton-insulated during freeze–thaw process, and the three groups of slopes were closely drained in parallel to prevent the influence of lateral temperature changes on the slope. All sensors are also located in the center of the slope in order to minimize the influence of temperature on the experimental results and the influence of the contact surface between the model box and the slope on the experimental results. As shown in Fig. 2, the displacement, temperature, moisture content and soil pressure positions monitored by the slope include the slope top, slope surface and slope bottom. At the same time, we also monitored the crack development on the slope surface.

The expansive soil maintains stable until the deformation rate of the soil exceeds 0.01 mm within 2 h. The test was divided into three stages: freezing at − 10 °C, freezing at − 20 °C and melting at 30 °C. The test specimens were basically stable for the first 6 days after being placed in the environment box at − 10 °C, and then, the temperature was decreased to − 20 °C for 3 days. Subsequently, the melting temperature of 30 °C was applied for 2 days. At the end of this 11-day process, one freeze–thaw cycle was completed. This experiment applied five freeze–thaw cycles in total.

## Results

### Crack development in the slopes

**Figure 3:**
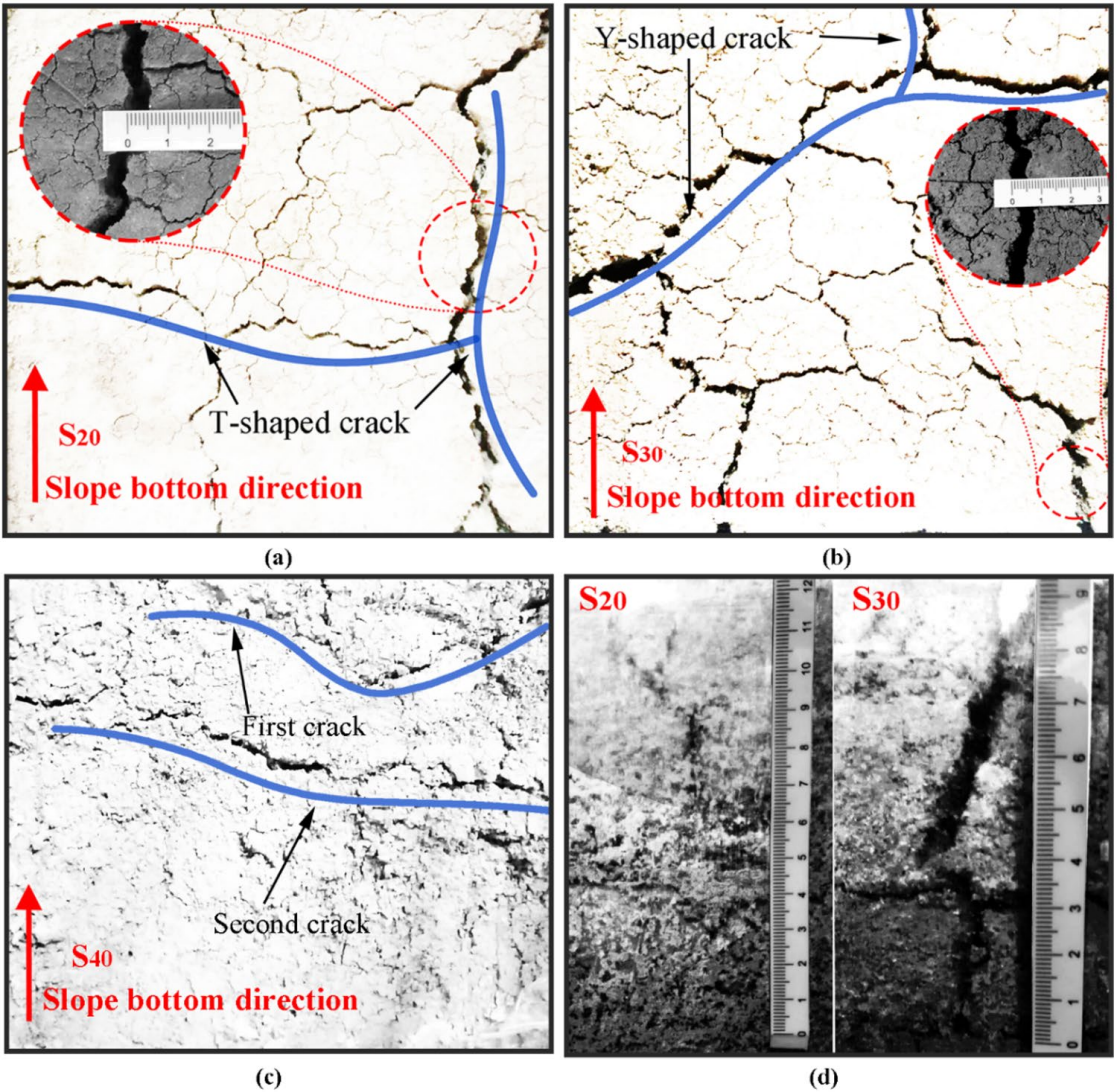
Cracks in the slope after 5 freeze–thaw cycles: cracks on the top of (**a**) $S_{20}$, and (**b**) $S_{30}$, and (**c**) $S_{40}$, and (**d**) cracks on the lateral side of $S_{20}$ and $S_{30}$.

Figure 3 shows the development process of cracks on the top of the three slopes with different moisture contents after five freeze–thaw cycles. A smaller T-shaped crack appeared on the top of the $S_{20}$ slope after the first freeze–thaw cycle. As the test proceeded, the width of the T-shaped cracks gradually increased. After the fourth freeze–thaw cycle, a longitudinal penetrating crack (main crack) appeared in the middle of the slope top. At the end of the fifth freeze–thaw cycle, the crack width continued to increase till 5 mm. The secondary cracks around the main crack were also obvious, and the number of cracks increased substantially, ultimately evolving into a network of cracks. A small inclined Y-shaped crack appeared at the top of the $S_{30}$ slope after the first freeze–thaw cycle, and the width of this Y-shaped crack increased continuously as the test elapsed. After the fourth freeze–thaw cycle, the cracks began to develop at the top edge of the slope. After the fifth freeze–thaw cycle, the slope surface was uniformly distributed with a large width, and the width of the cracks reached 5 mm. The cracks that were initially straight evolved to form polygonal cracks that basically covered the entire slope surface. The cracks at the top of the $S_{40}$ slope did not obviously develop during the five freeze–thaw cycles. Cracks did not appear on this slope until after the fourth freeze–thaw cycle. Moreover, these cracks did not develop from the edge position; instead, transverse short cracks appeared in the middle of the top of the slope, forming a straight line. After the fifth freeze–thaw cycle, another nonlinear crack appeared in the middle of the slope.

Figure 3d shows the cracks on the lateral side of two types of slopes after five freeze–thaw cycles. These lateral cracks formed due to the uneven contraction of the soil mass during freeze–thaw cycling^37^. The results show that when the moisture content decreased, the cracks became more developed along the depth direction. The crack depth in the $S_{20}$ slope body reached 9 cm after five freeze–thaw cycles, whereas that in the $S_{30}$ slope body reached 7 cm after five freeze–thaw cycles. In addition, no cracks could be observed on the side of the $S_{40}$ slope body after five freeze–thaw cycles.

### Soil pressure in the slopes during freeze–thaw cycling

**Figure 4:**
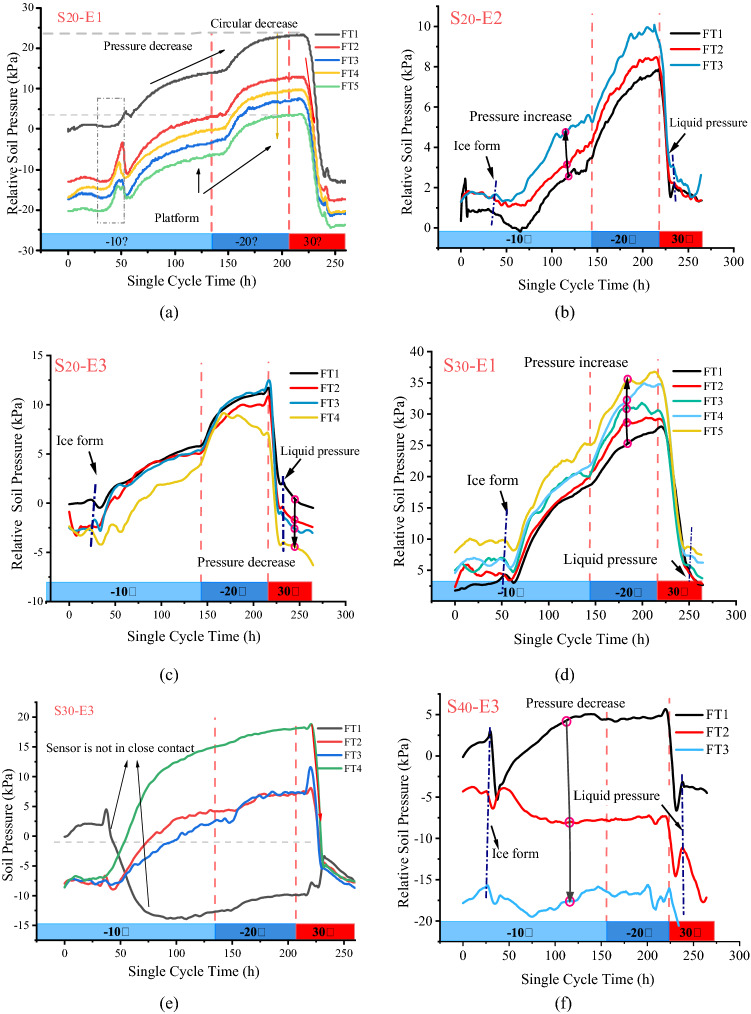
Relative soil pressure in the slope during multiple freeze–thaw cycles ((**a**) bottom of the $S_{20}$ slope; (**b**) middle of the $S_{20}$ slope; (**c**) top of the $S_{20}$ slope (**d**) bottom of the $S_{30}$ slope (**e**) top of the $S_{30}$ slope; (**f**) top of the $S_{40}$ slope).

Generally, the soil pressure in expansive soil slopes with different moisture contents changes during freeze–thaw cycling. Figure 4 shows the soil pressure changes in the $S_{20}$, $S_{30}$ and $S_{40}$ slopes during freeze–thaw cycling. In the slope model, sensors ***E1***, ***E2*** and ***E3*** were located at the bottom, middle and top of the slope, respectively. All soil pressure tests, such as Fig. 4b, shows the soil pressure increased during the freezing process and decreased during the melting process. During the freezing process for about 25 h, the soil pressure exhibited a short peak value and then decreased rapidly (all Fig. 4). Then the temperature of the frozen soil decreased to below 0 °C, and the free moisture in the soil greatly crystallized. The formation of ice-penetrating crystals and frozen fringes gradually caused the soil pressure to increase rapidly. The soil deformation gradually released the pressure. Later, the expansive soil lost moisture and contracted, and the soil cooled and contracted, so the soil pressure decreased for a short period. During the melting process, the soil temperature in the slope increased to 0 °C at approximately 240 h. A large amount of free moisture produced by ice melting caused an increase in soil pressure at the bottom of the slope. The excess pore moisture pressure dissipated, and the soil pressure was basically stable. During this process, a large amount of moisture was generated in a short time, which resulted in another short peak that were called “Liquid pressure” in Fig. 4b–d.

A comparison of the slope soil pressure during multiple freeze–thaw cycles is shown in Fig. 4, which reveals that the soil pressure at the top (***E3***, Fig. 4c) of the $S_{20}$ slope decreased during the freeze–thaw process, whereas the soil pressure at the bottom (***E1***, Fig. 4a) and middle (***E2***, Fig. 4b) of the slope increased. During freeze–thaw cycling, the soil structure became looser due to the moisture in the soil changing phase. However, the moisture migration between the slope and the bottom was substantial, which was associated with freeze–thaw consolidation. The two mechanisms together resulted in the soil pressure at the top of the slope decreasing during freeze–thaw cycling; the middle and bottom of the slope were more compacted, so the soil pressure increased during freeze–thaw cycling. In the test, different positions of the same slope have the same change, so the representative change pictures of each slope are selected in Fig. 4, and the follow-up monitoring data are displayed in the same way. It is worth noting that some of the soil pressure sensors were damaged during a long test process (44 days) and a very large temperature difference (40 °C), of which ${\text{S}}_{30}{\text{-E2}}$, ${\text{S}}_{40}{\text{-E1}}$ and ${\text{S}}_{40}{\text{-E2}}$ did not take two consecutive cycles of data, while others only monitored the changes in soil pressure in the first three cycles.

### Displacement changes in the slope during freeze–thaw cycling

**Figure 5:**
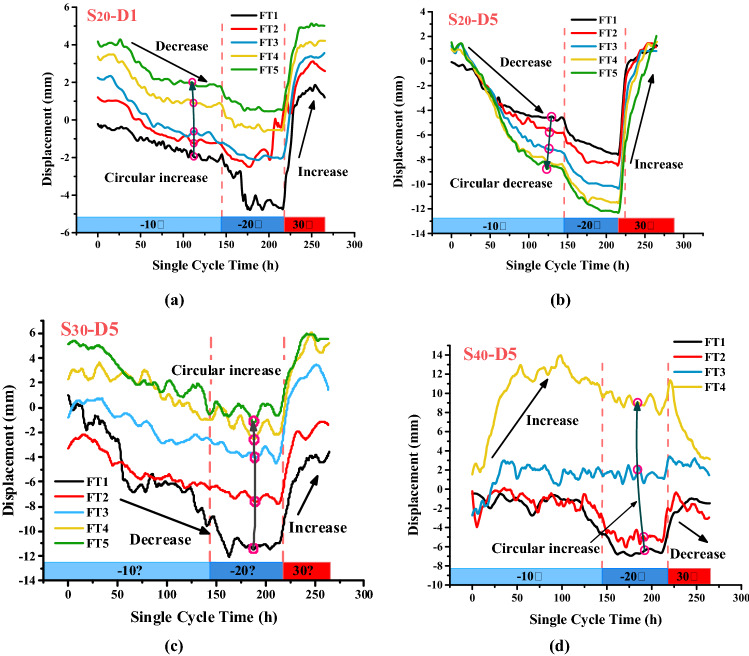
Slope displacement during freeze–thaw cycling ((**a**) bottom of the $S_{20}$ slope; (**b**) top of the $S_{20}$ slope; (**c**) top of the $S_{30}$ slope; (**d**) top of the $S_{40}$ slope).

Figure 5 shows the change in displacement in the slopes during freeze–thaw cycling, wherein the displacement of the $S_{20}$ slope model in a single freeze–thaw cycle decreased with decreasing temperature and increased with increasing temperature; this phenomenon is referred to as “freezing-shrinkage/thawing-expansion” (Fig. 5a, b). The top and middle of the slope exhibited a trend of progressive deformation away from the free surface. A single freeze–thaw cycle of the $S_{30}$ slope model also showed the phenomenon of “freezing-shrinkage/thawing-expansion,” but the overall volume increased, exhibiting a trend of progressive deformation toward the free surface. In the $S_{40}$ slope model, the “freezing-shrinkage/thawing-expansion” phenomenon appeared in the first two freeze–thaw cycles, whereas the subsequent freeze–thaw cycles all exhibited the “freezing-expansion/thawing-shrinkage” phenomenon and a trend of gradual deformation toward the free surface as a whole.

Freeze–thaw cycles have a greater impact on slopes with a higher moisture content. Hence, for the $S_{30}$ and $S_{40}$ slopes, multiple freeze–thaw cycles loosened their structures and increased their volume. However, for the $S_{20}$ slope, as the number of freeze–thaw cycles increased, its internal moisture loss decreased, and freeze–thaw settlement occurred on the surface of the slope. The cumulative changes from these two cycles were larger than the structural loosening and volume increase caused by freeze–thaw cycling, resulting in an overall volume reduction. Soil shrinkage in the $S_{20}$ and $S_{30}$ slopes during a single freezing process was caused by the loss of moisture and the shrinkage of expansive soil particles, associated with low-temperature shrinkage of the soil. These two changes were larger than the volume increase generated by ice crystals, and both together led to the freezing-shrinkage phenomenon of the expansive soil slope, which is named *ES-FSTE* (Expansive Soil Freezing-Shrinking and Thawing-Expansive) Model.

### Moisture content in the slope during freeze–thaw cycling

**Figure 6:**
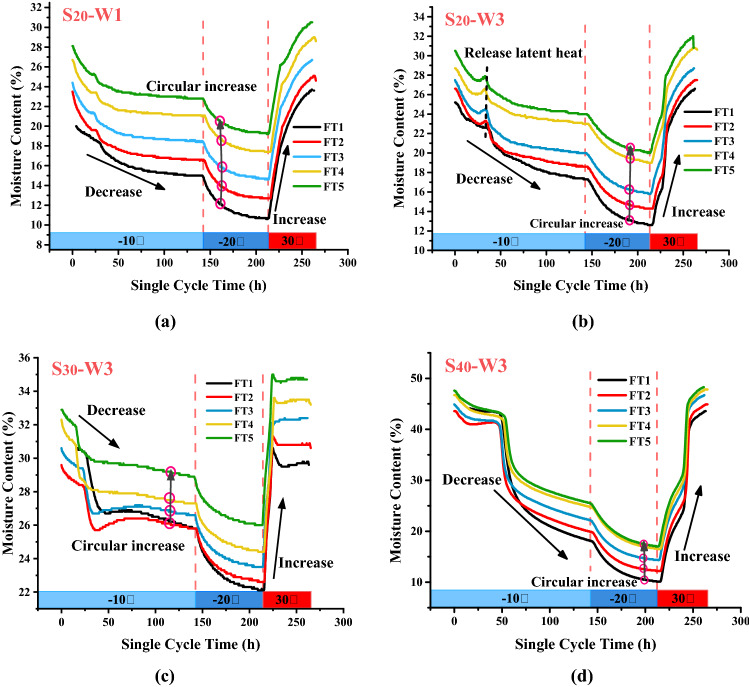
Moisture content in the slopes during freeze–thaw cycling ((**a**) bottom of the $S_{20}$ slope; (**b**) top of the $S_{20}$ slope; (**c**) top of the $S_{30}$ slope; (**d**) top of the $S_{40}$ slope).

Figure 6 shows the moisture content changes in the $S_{20}$, $S_{30}$ and $S_{40}$ slopes at different locations during freeze–thaw cycling. The moisture content change in the slope during a single freeze–thaw cycle can be divided into three stages: the cooling and freezing stage, the freezing and stabilizing stage, and the high-temperature thawing stage. The moisture content continued to decrease during the cooling and freezing stages. When the temperature of the slope surface decreased below 0 °C, the moisture content decreased mainly due to water–ice phase transition. When the external temperature decreased, the temperature variation gradually propagated from the surface to the slope body. Accordingly, the free water in expansive soil gradually changes into ice solid, resulting in a decrease in the moisture content of the slope surface^38^. the moisture in the slope migrated toward the lower temperature. During the freezing process, there is a small amount of moisture migration, which leads to the migration of water in the soil to the slope surface, but this migration is far less than the free water volume of phase transition, and finally leads to the overall performance reduction of moisture content. When the temperature reached 0 °C or below, the freezing of the moisture in the soil led to a continued decrease in the moisture content. When the temperature increased, the ice in the upper soil of the sensor melted before reaching 0 °C, and the moisture content continued to increase as the melting process continued. However, in the process of soil freezing, not all the moisture was transformed into ice. Due to the effect of particle surface energy, soil particles retained a certain amount of moisture, and the moisture content did not drop to 0 during freeze–thaw cycling. During multiple freeze–thaw cycles, it can be observed that the moisture content at the sensor position continued to increase, and there was an obvious upward migration phenomenon of the slope moisture.

A comparison of the moisture content changes in the different slopes showed that the moisture content in the $S_{30}$ slope changed the most after each freeze–thaw cycle, whereas the moisture migration in the slopes with a high moisture content was not obvious during multiple freeze–thaw cycles. Similarly, there was a transient peak at the beginning of the freezing process, when the temperature at the sensor location decreased to 0 °C, and a large amount of latent heat was released by the phase change of the free moisture, resulting in a slower temperature decrease and affecting the moisture content changes^39^. Figure 6 indicates that as the slope moisture content increased, the duration of this peak gradually increased, and even a short platform appeared in the $S_{40}$ slope. Due to the characteristics of expansive soils, the changes in the slope moisture content led to a volumetric contraction, which supports the interpretation of the slope volume changes in “Soil pressure in the slopes during freeze–thaw cycling” section.

### Loading test after freeze–thaw cycling

**Figure 7:**
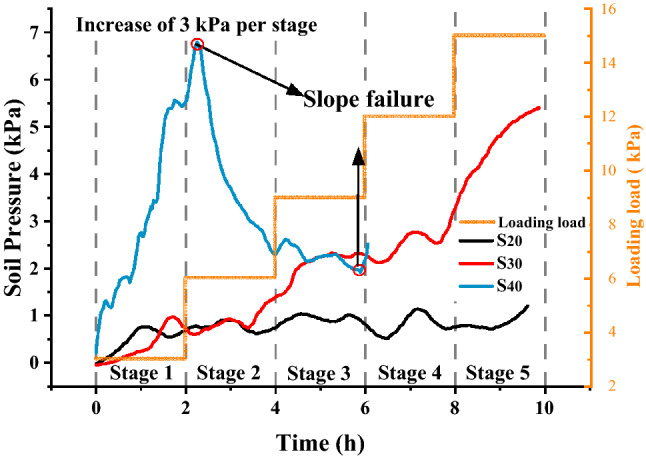
Soil pressure after five freeze–thaw cycles.

After five freeze–thaw cycles, a static load test was carried out on the test specimens. For this process, 0.09 m^2^ wooden boards were placed on the top of each slope, which was loaded in stages, wherein the loading in each stage was 3 kN/m^2^. Figure 7 shows the changes in soil pressure under step loading after the fifth freeze–thaw cycle. The soil pressure in the $S_{20}$ and $S_{30}$ slopes increased slowly. The soil pressure in the $S_{40}$ slope increased rapidly at first and then decreased rapidly. The soil pressure changes in the $S_{30}$ slope was small and gentle, and in the later stage, the soil pressure changes in the S_20_ slope were larger than those in the $S_{30}$ slope. The displacement changes in the slope under step loading are shown in Table 2. This is the same as the bearing capacity of the slope studied before. Increased water content will significantly reduce the bearing capacity of the slope and increase the deformation capacity of the slope, so that $S_{40}$ has been destroyed under the second stage load, and the increase of soil pressure and displacement of $S_{30}$ is significantly greater than $S_{20}$.

**Table 2:** Displacement of the slopes after step 5 loading.

Sensor	Relative displacement (mm)		
	$S_{20}$	$S_{30}$	$S_{40}$
***D1***	− 0.10	− 0.99	0.10
***D2***	0.37	0.21	− 0.10
***D3***	0.30	0.19	− 1.19
***D4***	0.88	0.98	1.99
***D5***	4.71	6.26	5.98

Note that the $S_{20}$ and $S_{30}$ slopes were subjected to eight loading stages, whereas the $S_{40}$ slope was subjected to four loading stages.

During the loading test, the normal displacement of the slope top increased as the moisture content in the slope increased. The average deformation of the $S_{20}$, $S_{30}$ and $S_{40}$ slopes after the loading test was 0.589 mm, 0.783 mm and 0.996 mm, respectively. Due to freeze–thaw cycling, the $S_{20}$ slope deviated from the free surface as a whole. Namely, the slope volume decreased, and the compactness increased. In contrast, the $S_{30}$ and $S_{40}$ slopes developed toward the free surface as a whole, and the density decreased, which led to a gradual increase in the deformation of each slope under the same load.

## Discussion

### Comparison with the one-dimensional frost-heaving model and ES-FSTE model

The expansive soil used in this study was clayey soil with a large amount of entrapped gas, which was isolated from the atmosphere in an unsaturated state. This entrapped gas directly affected the expansion properties of the soil. In particular, the hysteretic behavior of the clay was substantially affected by this entrapped gas. For clay, the time from initial deformation to reaching a stable state can be twice as long as that of sandy soil. Roman et al.^40^ used the following representation for the linear strains in solids, liquids, and gases in frozen soils according to thermodynamic principles:

1 $$\varepsilon_{i} = \alpha_{i} \Delta T(i = 1,2,3)$$

where $\varepsilon_{1}$ represents the solid strain, $\varepsilon_{2}$ represents the liquid strain, $\varepsilon {}_{3}$ represents the gas strain, $\alpha_{{\text{i}}}$ represents the corresponding one-dimensional linear strain coefficients, and $\Delta T$ represents the temperature change.

The volumetric strain in the soil with respect to temperature is not an accumulation of the three linear strains but rather the corresponding volumetric strain resulting from the stress redistribution due to mutual restraint of the three strains. The corresponding strain of the soil can be expressed as follows:

2 $$\varepsilon = \alpha \Delta T$$

where $\alpha$ is measured in a one-dimensional expansion experiment by the one-dimensional length variation in the specimen.

**Figure 8:**
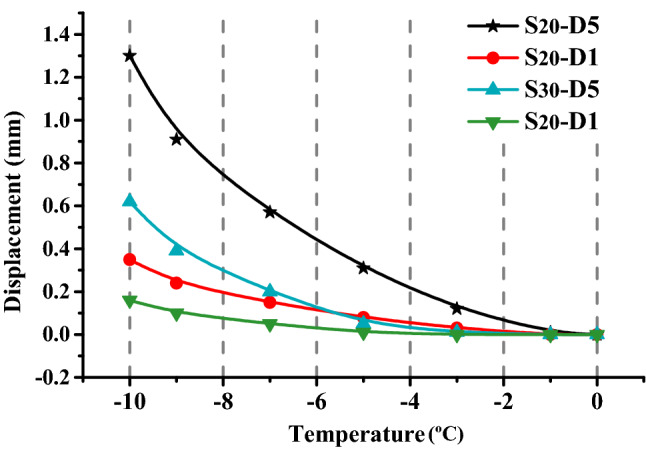
Displacement at 0–10 °C according to Roman’s frost-heaving model.

The measured one-dimensional coefficient of expansion (− 1 to − 9 °C) for the clay was interpolated to (0 to − 10 °C) on both sides at different moisture contents. The slope displacement during the first freeze–thaw cycle was calculated according to this coefficient of expansion α (Table 3). The results are shown in Fig. 8.

**Table 3:** Fitting the slope expansion coefficient with a one-dimensional expansion model.

Moisture content (%)	Frost-heaving coefficient	Temperature range (°C)					
		0 to − 1	− 1 to − 3	− 3 to − 5	− 5 to − 7	− 7 to − 9	− 9 to − 10
20	$\alpha \cdot 10^{5} ,\;\deg^{ - 1}$	2	20	35	47	61	71
30	$\alpha \cdot 10^{5} ,\;\deg^{ - 1}$	0	2	7.5	28	35	42

When the temperature was 0 to − 10 °C, the displacements at ***D1*** and ***D5*** in the $S_{20}$ slope were − 6.3 mm and − 2.9 mm, respectively, whereas these displacements in the $S_{30}$ slope were − 8.3 mm and − 1.5 mm, respectively. ES-FSTE model is contrary to the fitting results from the one-dimensional expansion model. In the first freezing process, the $S_{20}$ and $S_{30}$ slope models exhibited freezing-shrinkage, because the slope model actually exhibited two-dimensional expansion, and expansive soil will freeze and shrink when the moisture content is low (see “Displacement changes in the slope during freeze–thaw cycling” section). Extra attention needs to be paid to the freezing-shrinkage characteristics of expansive soil slopes under certain conditions. Unlike the common freezing- expansion model, the freezing-shrinkage and thawing-expansion in the slope will possibly increase the internal porosity and loosen the structure, which can potentially cause engineering disasters. In the conventional freeze–thaw model, the porosity of the slope increases during freeze–thaw cycling; but the freeze–thaw consolidation effect will reduce the porosity of the slope during freeze–thaw cycling. However, in the freezing-shrinkage and thawing-expansion model, the freezing-shrinkage and drying-shrinkage of the soil mass is still associated with an increase in soil porosity. But when the soil expands, due to the strong moisture absorption of expansive soil particles the expansive soil particles absorb free moisture, and the freeze–thaw consolidation effect is not obvious, resulting in slope soil increase the internal porosity and loosen.

### Changes in the expansive soil slopes during freeze–thaw cycling

#### Crack development in the slopes

**Figure 9:**
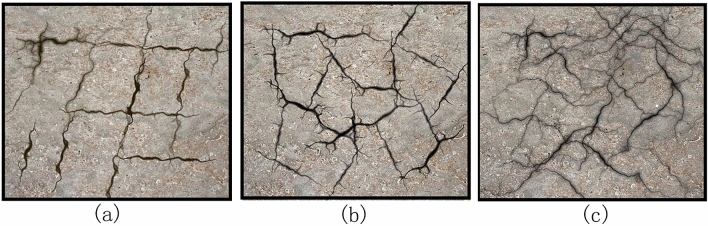
Schematic diagram of crack classification in three different states ((**a**) networked cracks, (**b**) polygonal crack, and (**c**) random cracks).

The expansive soil slope model shows obvious crack development on the slope surface during freeze–thaw cycling, which is important evidence of soil permeability and structural strength changes. The degree of fracturing of the expansive soil slope are revealed by the development of cracks. The geometric characteristics of the cracks should be considered and classified from these observations (Fig. 9). According to their orientation, the cracks can be divided into networked cracks, polygonal cracks and random cracks^41^. As shown in Fig. 3, the $S_{20}$ slope has two groups of approximately vertical cracks, which can be classified as networked cracks; the $S_{30}$ slope gradually evolves into a network of main cracks and secondary cracks during freeze–thaw cycling, and these cracks can be classified as polygonal cracks; and the $S_{40}$ slope does not exhibit any obvious crack development phenomenon. The development of cracks in expansive soil slopes under freeze–thaw cycling is closely related to the moisture content in the slope, and the lower the moisture content, the more obvious the development of cracks. In general, the shape of cracks gradually changes from regular to irregular with the increase of moisture content. Cracks of different depths and widths can provide external water sources with a path into the interior of the slope, thus indirectly changing the permeability of the slope, in other words, the permeability of the slope is proportional to the degree of crack development of the slope.

The lateral and longitudinal cracks in the slopes after freeze–thaw cycling divide the soil mass into related units with large relative displacements. When calculating the slope stability, the calculation units can be approximately selected according to the characteristics of crack development. The crack development in the units of the slope was more regular when the moisture content was lower. With increasing moisture content, the cracks in the slope developed into irregular polygons. When the slope reached a high-moisture content, only a few cracks occurred, and the change in the slope body was more integrated. Micro-analysis of the origins of different cracks are discussed in “Structural changes in the expansive soil slopes” section. During the test, we found that the crack development of expansive soil has changed greatly with the change of moisture content. When mitigating engineering disasters of expansive soil, some scholars used crack filling^18^ to increase the strength of expansive soil, and some scholars also used drip irrigation^19^ to restrain the formation of cracks in expansive soil. Therefore, the crack development of expansive soil slope under different moisture content can be used for these improvements. Provide basis and provide effective help for freeze–thaw modeling of expansive soil slope.

#### Structural changes in the expansive soil slopes

The changes in slope displacement during freeze–thaw cycling (Fig. 5) show that all three slopes with different moisture contents exhibit “freezing-shrinkage and thawing-expansion” during the first freeze–thaw cycle. The $S_{20}$ and $S_{30}$ slopes exhibited this phenomenon throughout the testing process, whereas the $S_{40}$ slope exhibited “freezing-expansion and thawing-shrinkage” in the later freeze–thaw cycles. Due to the characteristics of moisture absorption, expansion and shrinkage of expansive soils, the freezing process is associated with moisture loss and shrinkage during freeze–thaw cycling, and the melting process is associated with moisture absorption and expansion, which is opposite to the frost-heaving and thawing characteristics of ordinary low expansion clays during freeze–thaw cycling. Freezing–thawing is a special coupling effect on expansive soils, which can be divided into the increase of volume from water to ice, and the shrinkage of expansive soils due to the reduction of free water in the expansive soils, which has a similar effect in the melting stage. Because of the complexity of displacement of expansive soil during freeze–thaw, the displacement change of expansive soil during freeze–thaw is significantly different from that of traditional frozen clay.

From the macroscopic point of view, for expansive soil slopes with lower moisture content, the volumetric expansion from moisture crystallization cannot offset the volumetric shrinkage from moisture loss, which will cause pore loosening in the expansive soil. For expansive soil with low moisture content, the expansive soil slopes contracts during the freezing stage. Similarly, the moisture absorption and volumetric expansion of the expansive soil during melting is lower than those during freezing. Accordingly, the volume of the whole slope body expands. The above phenomenon is caused by the volume change of expansive soil particles and moisture in soil during freezing and thawing. It is well known that changing water to ice during freezing results in an increase in volume and a decrease in volume when ice thaws to water. Conversely, the major component of expansive soils, layered silicates, has a significant volume decrease during frozen water loss process and has a large volume increase during melted water absorption. Therefore, the volume change of water and expansive soil particles is opposite in freezing and thawing, while the volume change of expansive soil depends on the relative content of both, i.e., moisture content. From the microscale point of view, the displacement of the slope varies greatly during freeze–thaw cycling because each freeze–thaw cycle will induce strong structural changes, including changes in the skeleton and pores of the expansive soil.

**Figure 10:**
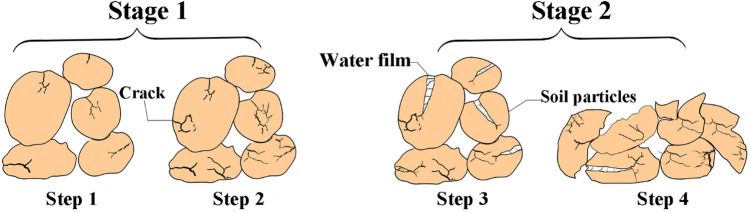
Schematic diagram of the soil weathering process during freeze–thaw cycling.

The soil skeleton includes soil particles, gums and combined moisture. Contact between the soil particles in the soil skeleton directly affects the mechanical properties of the soil. As contact areas between the soil particles in the soil skeleton increase, the skeleton becomes denser and more uniform in the process of force transmission^42^. Different moisture contents in the soil affect the thickness of the moisture film on the surface of the soil particles, and then alter affecting the friction and deformation capacity between soil particles. As an especially strong weathering process, freeze–thaw cycles cause moisture migration and phase changes in the slope through temperature changes. The freeze–thaw weathering process of soil mineral particles can be divided into two stages. In the first stage, the volume of moisture increases by approximately 9% during freezing^43^. The damage of freeze–thaw cycle to soil particles can be divided into four steps. The moisture-to-ice phase change in the soil causes cracks on the surface of the mineral particles (Fig. 10, Step 1). The microcracks develop into macrocracks as the particles undergo freeze–thaw cycling (Fig. 10, Step 2). In the second stage, the moisture content in the particulate cracks of the soil increases (Fig. 10, Step 3), resulting in further crushing of the primary minerals. Finally, under the frost-heaving force of the moisture, the soil particle breakage develops toward homogenization (Fig. 10, Step 4). The primary minerals are fragmented and reassembled under freeze–thaw cycling, which changes the skeleton structure, homogenizes soil particles and makes the interaction between soil particles less intense^44^. The development of soil particle breakage and homogenization in expansive soil slopes will lead to the enlargement of the contact area between soil particles and moisture, which will strengthen the characteristics of moisture absorption expansion and moisture loss contraction, causing the S_20_ slope to show the characteristics of “freezing-shrinkage and thawing-expansion.”

**Figure 11:**
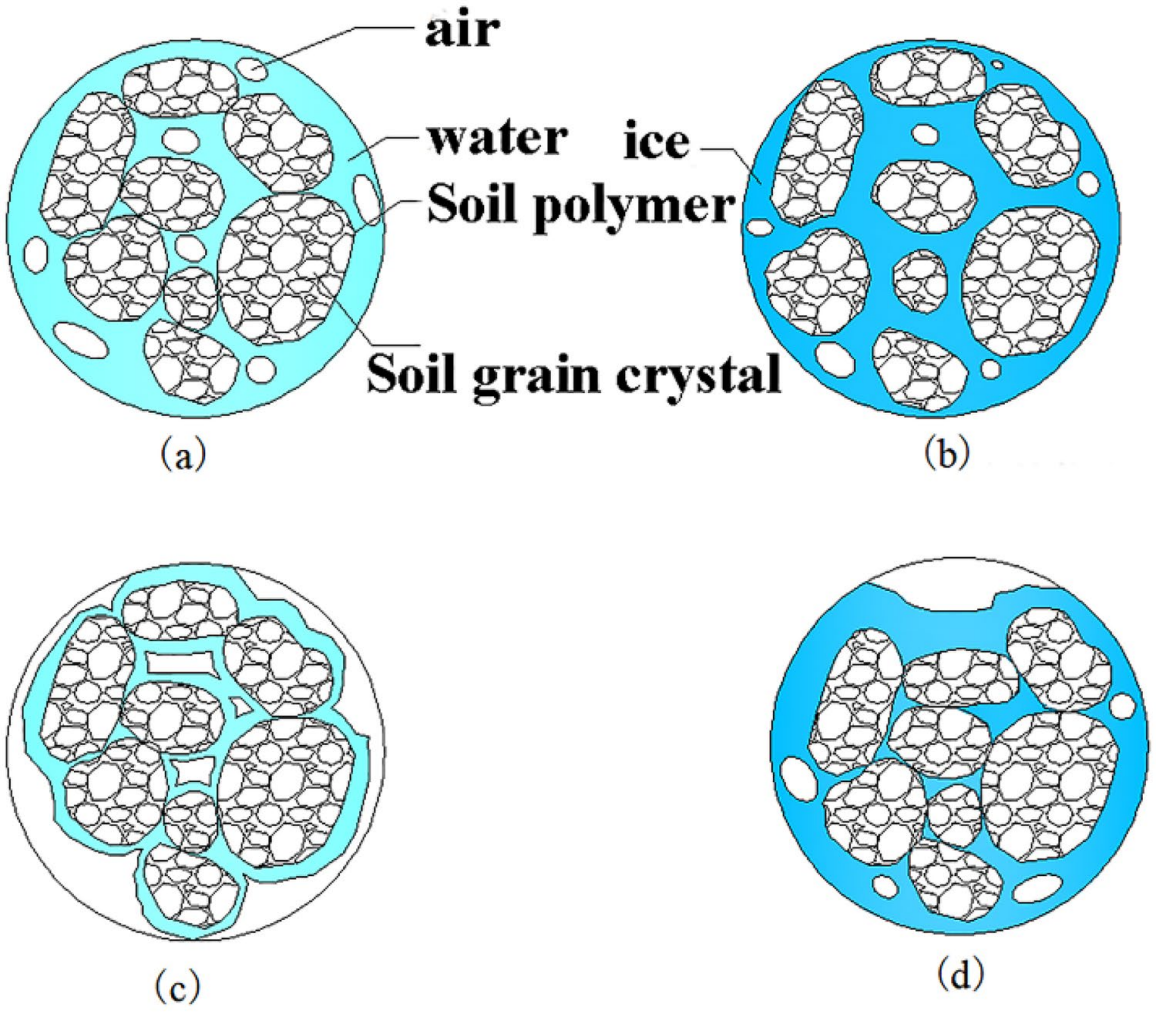
Pore changes in the soil during freeze–thaw cycling. (**a**) High moisture content (unfrozen); (**b**) high moisture (frozen); (**c**) low moisture (unfrozen); (**d**) low moisture (frozen).

Figure 11 shows a sketch of the pore changes in expansive soil with different moisture contents after freezing. The porosity of soil shows two changes under the two states of high moisture content and low moisture content. Figure 11a and c illustrate the three-phase composition and distribution of unfrozen expansive soils with high and low moisture contents, respectively. As described by Sanchez et al.^45^, expansive soils usually show obvious dual porosity with two main pore sizes. There is an internal micropore distribution between clay grain crystals. Clay crystal particles aggregate to form aggregates, and macropores are formed between these aggregates.

When the soil with high moisture content is frozen, as shown in Fig. 11, the pore space between the aggregates is occupied by moisture that is changing phase into ice, which gradually enlarges the pore space between the aggregates and fills the macropores with expanding ice. Moreover, the formation process of ice crystals can produce surface pressures ranging from 100 to 460 kPa, where in the ice crystals growing in the aggregates and crystal pores compress the soil aggregates and crystal particles. Under the influence of the expansion pressure generated by the ice, the rearrangement of some particles will occur when some aggregate pore moisture freezes^46^. At the same time, due to the low temperature, the moisture inside the particles migrates outwards, which leads to drying- shrinkage cracks inside the aggregate^47,48^. The local transfer of moisture between clay crystals and structural moisture into the macropores of the soil will lead to secondary drying-shrinkage of the clay^49^. The volume of the frozen soil exhibits freezing-shrinkage due to the dramatic changes in moisture absorption expansion and moisture loss shrinkage of the expansive soil particles.

Generally, during the melting stage, the ice changes phase into moisture, which causes the volume to shrink. The expansive soil particles absorb moisture, causing expansion, but the moisture absorption expansion of the soil particles is much smaller than the volumetric shrinkage produced by the ice melting, which results in the rearrangement of the soil particles and the volumetric shrinkage of the soil as a whole. However, the microstructure of the soil with a low moisture content during the freezing process, which is shown in Fig. 11d, reveals that the existing air in the pores during the freezing process adequately accommodates the volumetric expansion caused by the phase change of moisture to ice between the aggregates. In this case, the volumetric shrinkage effect is related to the shrinkage of the soil particles. If the initial pore gas volume is sufficient to accommodate ice expansion, the volumetric shrinkage of the soil particles will be greater than the expansion caused by the moisture in the soil particles changing phase into ice, and freezing-shrinkage occurs in the soil body. During the melting phase, the internal temperature and temperature gradients cause moisture to migrate from the internal pores of the aggregate to the internal pores of the soil grain crystals. Due to hysteresis, the soil retains more moisture at low temperatures than during melting. Therefore, the soil particles no longer expand to their original volume as a result of moisture absorption. In addition, due to irreversible plastic deformation caused by the ice, large voids remain between the aggregates.

#### Moisture migration in the slopes

Moisture molecule exists in ice, moisture, and steam phases in the slope model, and there is a moisture migration phenomenon in the expansive soil slope during freeze–thaw cycling (Fig. 2). In Fig. 6, it can be observed that the moisture content of the expansive soil slope increased after multiple freeze–thaw cycles, and the moisture content at different depths showed an obvious moisture migration phenomenon. With increasing freeze–thaw cycles, the moisture content at ***W3***, ***W4*** and ***W5*** increased as a whole, indicating that moisture migration occurred. During the freezing process, the temperature of the soil slope decreased, the frozen front moved downward, and the upper soil froze, whereas the unfrozen moisture in the lower soil moved upward under the influence of a temperature gradient, resulting in higher moisture content at a shallower depth in the slope. As shown in Fig. 6, the fluctuation range in moisture content increases with the increase of slope model moisture content during freeze–thaw cycling.

**Figure 12:**
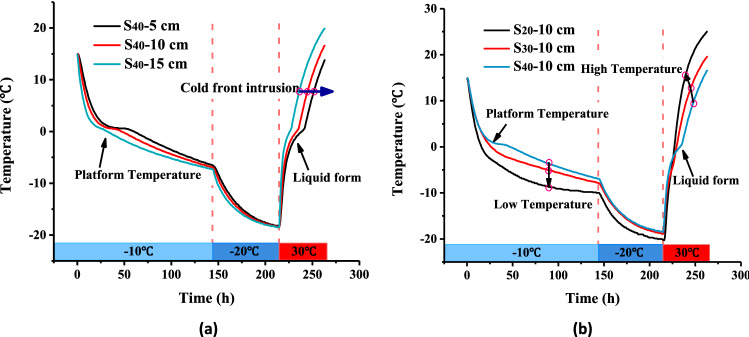
Temperature variation during a single freeze–thaw cycle at (**a**) different depths in the S_40_ slope and in (**b**) three slopes at a depth of 10 cm.

During the freezing process of the slope, the cold front intruded from the slope surface, and moisture accumulated on the slope surface due to its migration toward lower temperature side and then moved back during the melting process. The increased moisture content in the slope indicated that the moisture migration to the slope surface during the thawing process was less than that from the freezing process, which was affected by the physical properties of the expansive soil and the rate of freezing. The freezing process is actually a process of the cold front intruded into the slope model, resulting in frost-heaving or freezing-shrinkage of the slopes. This process was associated by a change in the dry density of the slope. For expansive soil under the same moisture content, the soil and moisture potential decreased with decreasing dry density. In the melting stage of the slope, the internal temperature reached a homogeneous state relatively quickly. Hence, the rate of temperature increase in the melting stage was greater than the rate of temperature decreases in the freezing process (Fig. 12), which led to relatively small changes in the dry density of the slope during the melting stage and a smaller amount of moisture migration.

The development trend of the $S_{20}$ slope deviated from the free surface after freeze–thaw cycling, which was caused by the joint action of freeze–thaw consolidation of the slope and moisture loss and shrinkage of the expansive soil during moisture migration. There were drastic changes in the moisture content at different depths of this slope during freeze–thaw cycling. This process of moisture migration led to freeze–thaw consolidation of the expansive soil particles. Moreover, the structural changes in the expansive soil particles discussed in “Structural changes in the expansive soil slopes” section also indicate that the expansive soil slope tended to exhibit freezing-shrinkage, whereas the change in volumetric expansion during thawing was not as great as that of freezing-shrinkage. These results in a difference from the traditional concept of frost expansion and thawing shrinkage of soils. On the contrary, with the increase of freeze–thaw cycles, the expansive soil slope with low moisture content receives the influence of shrinkage of expansive soil particles and will develop towards the direction below the original slope surface. The freezing shrinkage of expansive soil in displacement monitoring will also lead to this result.

### Bearing capacity of the slope after freeze–thaw cycling

**Figure 13:**
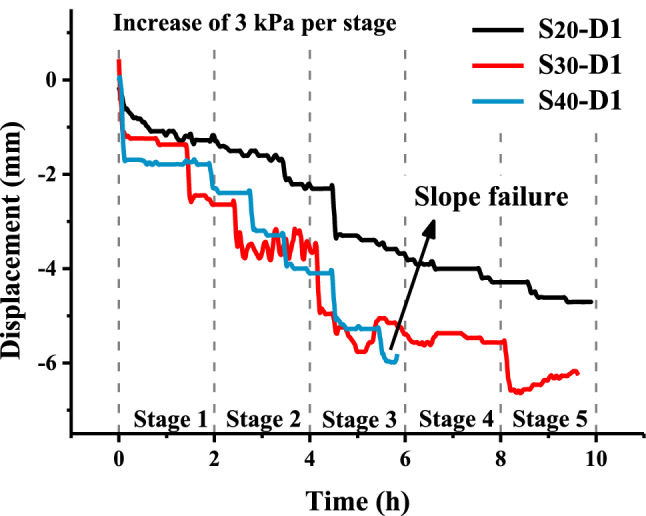
Displacement of the slope after freeze–thaw cycling.

After five freeze–thaw cycles, the deformation of the slope model increased with increasing moisture content, and the $S_{40}$ slope could not withstand the eight graded loading stages. The change in moisture content and displacement was the highest in the $S_{40}$ slope during freeze–thaw cycling. The continued expansion and shrinkage of soil particles in different freeze–thaw stages results in larger internal voids in the soil skeleton of the slope model, and this looser soil structure substantially reduced the bearing capacity. For expansive soils with a lower moisture content, consolidation caused by moisture migration leads to different dry densities of the upper and lower layers of the slopes and a smaller displacement while bearing greater loads. As shown in Fig. 13, for the $S_{30}$ and $S_{40}$ slopes, the load bearing capacity was poor after freeze–thaw cycling, and the deformation of the slope was greater when loading. This indicated that the bearing capacity and stability of expansive soil slopes with a high moisture content was poor after freeze–thaw cycling. There are similar conclusions in the study of the stability of expansive soil slopes^20,50,51^.

## Conclusion

1. During freeze–thaw cycling, cracks in expansive soil slopes developed from linear cracks with smaller widths to networked cracks with larger widths. The depth of lateral cracks developed grows as the initial moisture content of the soil slope decreases.
2. The difference between the volumetric changes in the soil particles and moisture in the melting and freezing process varies with different initial moisture contents within the soil slopes, which leads to soil pressure increase at the bottom soil slope pressure and decrease at the top of soil slope after freeze–thaw cycling. During the freeze–thaw process, as the initial moisture content of the slopes increase, the changes in the slopes soil pressure increase.
3. The moisture migration was associated by freeze–thaw process in the slope, and as the moisture content increases, the amplitude of moisture content change increases. Moisture migrates upward and downward in freezing processes and melting processes, respectively, and consequently, the moisture content in the upper layer of the slope is increased. The moisture content becomes progressively stabilized as the number of freeze–thaw cycles increases.
4. Under freeze–thaw cycling, the expansive soil slope with a low moisture content shows “freezing-shrinkage and thawing-expansion” and deviates from the free surface, whereas the slope with a high moisture content exhibits “freezing-expansion and thawing-shrinkage” and migrates towards the free surface, and freezing-shrinkage and thawing- expansion model can lead to a looser slope.
5. Freeze–thaw cycling substantially change the internal skeleton structure of the expansive soil slope, resulting in an increased variation in displacement per loading stage as the moisture content increases. Slopes with a low moisture content can bear greater loads, whereas slopes with a high-moisture content are prone to large displacements during loading.

## Data Availability

All data are either published within this manuscript and its supplementary information or available on requests.
